# Gut Microbiome Variation Along A Lifestyle Gradient Reveals Threats Faced by Asian Elephants

**DOI:** 10.1016/j.gpb.2023.04.003

**Published:** 2023-04-22

**Authors:** Chengbo Zhang, Zhenghan Lian, Bo Xu, Qingzhong Shen, Mingwei Bao, Zunxi Huang, Hongchen Jiang, Wenjun Li

**Affiliations:** 1 Engineering Research Center of Sustainable Development and Utilization of Biomass Energy, Ministry of Education, School of Life Sciences, Yunnan Normal University, Kunming 650500, China; 2 State Key Laboratory of Biocontrol, Guangdong Provincial Key Laboratory of Plant Resources and Southern Marine Science and Engineering Guangdong Laboratory (Zhuhai), School of Life Sciences, Sun Yat-sen University, Guangzhou 510275, China; 3 Xishuangbanna National Nature Reserve Management and Protection Bureau, Jinghong 666100, China; 4 Asian Elephant Provenance Breeding and Rescue Center in Xishuangbanna, Jinghong 666100, China; 5 State Key Laboratory of Biogeology and Environmental Geology, China University of Geosciences, Wuhan 430074, China

**Keywords:** Asian elephant, Gut microbiome, Phylogenetic analysis, Lifestyle, Host health

## Abstract

The **gut microbiome** is closely related to host nutrition and health. However, the relationships between gut microorganisms and host **lifestyle** are not well characterized. In the absence of confounding geographic variation, we defined clear patterns of variation in the gut microbiomes of **Asian elephants** (AEs) in the Wild Elephant Valley, Xishuangbanna, China, along a lifestyle gradient (completely captive, semicaptive, semiwild, and completely wild). A **phylogenetic analysis** using the 16S rRNA gene sequences highlighted that the microbial diversity decreased as the degree of captivity increased. Furthermore, the results showed that the bacterial taxon WCHB1-41_c was substantially affected by lifestyle variations. qRT-PCR analysis revealed a paucity of genes related to butyrate production in the gut microbiome of AEs with a completely wild lifestyle, which may be due to the increased unfavorable environmental factors. Overall, these results demonstrate the distinct gut microbiome characteristics among AEs with a gradient of lifestyles and provide a basis for designing strategies to improve the well-being or conservation of this important animal species.

## Introduction

The gut microbiota is closely related to the nutrition and health of mammals, contributing to physiological processes [1], immune responses [2], and nutrient metabolism [3]. In particular, herbivorous mammals rely on their specific gut microbiota to extract nutrients and energy from food, synthesize vitamins, and detoxify plant defense compounds [4]. Normal microbe–host relationships are maintained by a healthy and stable diet [5], geographically homogeneous environments [6], and long-term continuous and nonfluctuating lifestyles [7]. Disturbances in these relationships can cause host illness [8]. However, it is difficult to separate the contributions of diet and geographic factors from lifestyle factors [7]. Therefore, understanding how and to what extent lifestyle affects the gut microflora remains a great challenge.

Numerous studies have shown that changes in animal lifestyle greatly alter the intestinal microflora [9], [10]. When wild animals are placed in captivity, they have to cope with drastic changes, such as a restricted range of activities, a monotonous diet, and the loss of specific social networks. These conditions interfere with the diversity and composition of the gut microbiota in animals [11]. Compared with wild animals, their captive counterparts have lower gut microbial diversity [12]. Nevertheless, conflicting results have been obtained regarding the differences in intestinal microbial communities between mammals in captivity and in the wild; for example, one study discovered that intestinal microbial diversity in captive rhinoceri was higher than that in wild rhinoceri [10]. These controversial results emphasize the need for more in-depth controlled studies on the impact of different lifestyles on animal gut microbiota [13].

It is helpful for wildlife protection to understand the interaction between host lifestyle and the gut microbiota composition. For example, habitat degradation leads to a decrease in the number of genes related to butyrate production and hydrogen metabolism in the gut microbiota of black howler monkeys, which may affect their health [14]. In captive folivorous primates, microbial diversity is reduced, leading to down-regulation of metabolic pathways related to fiber degradation [12]. Fibrolytic processes of the gut microbiota contribute up to 31% of the daily energy required by howler monkeys via the formation of short-chain fatty acids [15]. Accordingly, alterations in the gut microbial diversity and composition in response to shifts in lifestyle may have consequences for wildlife nutrition and health.

The Asian elephant (*Elephas maximus*, AE) is listed as a national first-level key protected wild animal in China. It is an endangered species according to the International Union for the Conservation of Nature and Natural Resources (IUCN). Wild Asian elephants (WAEs) are distributed in southern Yunnan Province, China, with a population ranging from 288 to 338 [16]. After decades of efforts to save and protect AEs, the Wild Elephant Valley tropical rainforest scenic area (> 2800 m^2^) includes animals with a gradient of lifestyles (details in sample description): completely captive (Cc), semicaptive (Sc), semiwild (Sw), and completely wild (Wi). Therefore, AEs in this area are suitable for research on the responses of intestinal microorganisms to lifestyle changes. However, comparative analyses of the gut microbiomes among animals with each of the abovementioned lifestyles are rarely reported.

In this study, based on the investigation of foraging plant species of AEs, we used bacterial community analysis (16S rRNA gene amplicon sequencing) to characterize the gut microbiome of AEs along a lifestyle gradient. Recently, the range of WAEs has gradually expanded northward from the Xishuangbanna area in southern China. Although this expansion has attracted global attention, the reason remains unclear. The specific questions that we seek to answer in this study include: (1) how does the gut microbiome of AEs vary along a lifestyle gradient in the Wild Elephant Valley? (2) which bacterial taxa in the AE microbiome are clearly influenced by the lifestyle gradient? (3) how does the content of genes related to host nutrition and health in the gut microbiome of AEs vary along the lifestyle gradient? The exploration of these questions will provide a basis for the design of strategies to improve the well-being or conservation of this important animal species.

## Results

### The diversity of plants foraged by AEs across the four lifestyles decreased with the degree of captivity

Based on a survey on plants foraged by AEs, only three types of food were supplied to Cc elephants, mainly consisting of elephant grass. In addition to being fed mainly elephant grass, Sc elephants also ate approximately 25 types of plants in the wild. The most frequently foraged plants were crabgrass (*Digitaria sanguinalis*) and bamboo leaves from the family Gramineae. Sw elephants foraged 49 species of plants in the wild, among which crabgrass and bamboo leaves were eaten most often. Approximately 112 species of plants were foraged by WAEs (Table S1). These results suggest that the diversity of plants foraged by AEs across the four lifestyles decreased with the degree of captivity. Although the diets of WAEs were highly diverse, they were extremely picky eaters. WAEs preferentially forage crops and cash crops, followed by bamboo shoots and young leaves, and finally hairy crabgrass and elephant grass. Based on the main foraged plants mentioned above (Table S2), we found that the average neutral detergent fiber (NDF) content increased with the degree of captivity (49.5% for Wi, 59.8% for Sw, 66.3% for Sc, and 72.8% for Cc; Table S3). However, the average crude protein (CP) contents were 8.5%, 14.93%, 12.1%, and 9.2% in the diets of Wi, Sw, Sc, and Cc elephants, respectively (Table S3). Typically, the higher the CP content and the lower the NDF content, the higher the nutritional value of forage [17], [18]. Based on the CP and NDF contents of foraged plants alone, the nutritional value of food in the Sw group may be superior among the four different lifestyles.

### Sex and age had no significant effect on the gut microbiome of AEs

A total of 2029 operational taxonomic units (OTUs) and 3,207,598 sequences [mean ± standard deviation (SD): 48,600 ± 5086.552; range: 35,650–61,086] were obtained from the fecal samples of 33 AEs (20 healthy AEs that did not receive antibiotics and 13 wild individuals). Rarefaction curves indicated near-saturation of community coverage (Figure S1), suggesting that the sequencing depth accurately reflects the bacterial community composition in all samples.

Based on results of the alpha diversity analysis [richness (Chao1index) and diversity (Shannon index)] at the OTU level (Figure S2A) and the beta diversity analysis [Permutational multivariate analysis of variance (PERMANOVA); *R*^2^ = 0.1771, *P* = 0.80, Figure S2B], there were no significant differences in the microbial diversity between the sexes. Similarly, no significant differences in the richness (Chao1 index) and diversity (Shannon index) of the gut microbiome (Figure S2C) or in the gut microbial composition (PERMANOVA; *R*^2^ = 0.1502, *P* = 0.098, Figure S2D) between juveniles and adults were observed. Therefore, data from males and females as well as juveniles and adults were pooled for subsequent statistical analyses.

### The alpha diversity of the gut microbiome of AEs decreased with the degree of captivity

**Figure 1:**
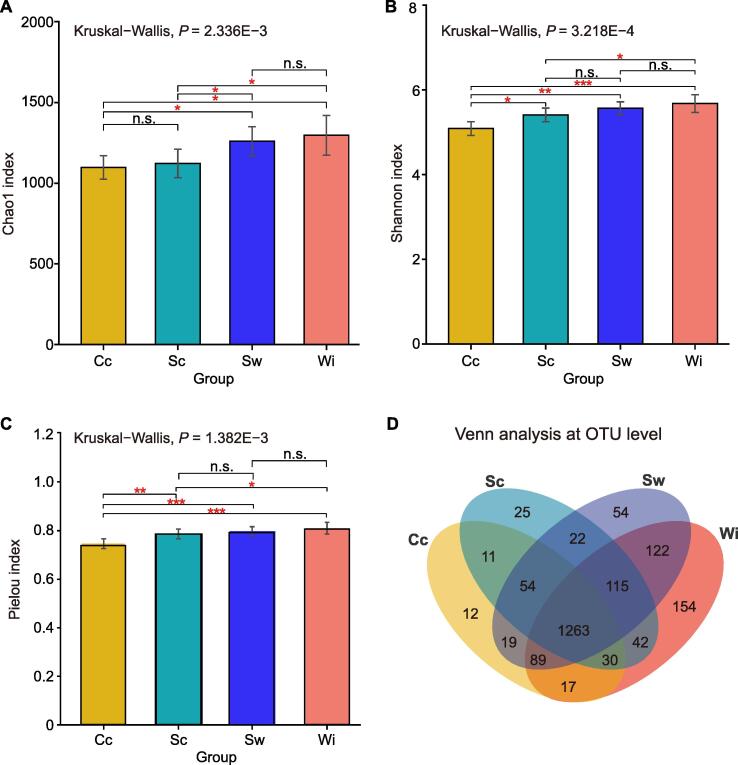
**Alpha diversityand Venn analysesof the gut microbiomesof AEsacross the lifestyle gradient** Chao1 (**A**), Shannon (**B**), and Pielou (**C**) indices of the gut microbiomes of AEs across four lifestyles. **D.** Venn analysis of the gut microbiomes of AEs across four lifestyles. *, *P* < 0.05; **, *P* < 0.01; ***, *P* < 0.001; n.s., no significance (Wilcoxon-Mann-Whitney test). AE, Asian elephant; Cc, completely captive; Sc, semicaptive; Sw, semiwild; Wi, completely wild.

The alpha diversity of the gut bacterial community of AEs decreased significantly with the degree of captivity (in the order of Wi, Sw, Sc, and Cc lifestyles; Kruskal−Wallis test, *P* < 0.05; Figure 1). The richness (Chao1 index) of the intestinal bacterial community clearly decreased from wild to captive lifestyles (Figure 1A), and the richness values for the Cc and Sc lifestyles were significantly lower than those for the Sw and Wi lifestyles (Wilcoxon-Mann-Whitney test; *P* < 0.05). In addition, the gut bacterial community diversity (Shannon index) of the AEs clearly decreased from the wild to captive lifestyles, with significant differences between the Cc and other three lifestyles (Wilcoxon-Mann-Whitney test; *P* < 0.05) and between the Sc and Wi lifestyles (Wilcoxon-Mann-Whitney test; *P* < 0.05) (Figure 1B). The evenness (Pielou index) of the gut bacterial community clearly increased from Cc to Wi lifestyles (Figure 1C). The evenness value for the Cc lifestyle was significantly lower than those for the other three groups (Wilcoxon-Mann-Whitney test; *P* < 0.01, Figure 1C), indicating that there were extremely dominant microorganisms in the individuals with a Cc lifestyle, which is consistent with the mono-diet of Cc elephants. There were 122 OTUs shared in the Sw and Wi groups that were not available in the Cc and Sc groups (Figure 1D). Among them, *Erysipelatoclostridium*-related OTU975, *Treponema* 2-related OTUs (OTU590 and OTU1438), Ruminococcaceae *UCG-005*-related OTU1382, Lachnospiraceae-related OTU471, and Mollicutes *RF9*-related OTU542 were separately present in more than half of the samples of the Sw and Wi groups. These microbiota were adapted to the diet of WAEs. There were 54 OTUs shared in the Cc, Sc, and Sw groups that were not found in the Wi state under human interference (Figure 1D). Among them, Bacteroidales_BS11-related OTUs (OTU222, OTU271, and OTU1183), *Prevotella* 1-related OTU1, Lachnospiraceae-related OTU194, Leptospiraceae-related OTU253, and *Pyramidobacter*-related OTU1281 were persent in more than half of the samples of the three groups. These microbiota were associated with the high-fiber diets of captive AEs.

### Artificial feeding might lead to the loss of bacterial taxa

**Figure 2:**
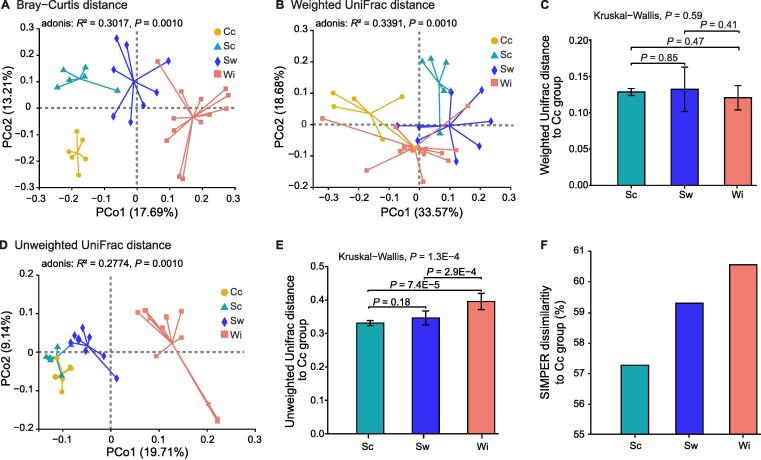
**Beta diversity analysis of the gut microbiomesof AEsacross the lifestyle gradient** **A.** Beta diversity analysis of the gut microbiomes of AEs across four lifestyles based on Bray–Curtis distances. **B.** Beta diversity analysis of the gut microbiomes of AEs across four lifestyles based on weighted UniFrac distances. **C.** Weighted UniFrac distances of the free-foraging lifestyles (Sc, Sw, and Wi) to the Cc group. **D.** Beta diversity analysis of the gut microbiomes of AEs across four lifestyles based on unweighted UniFrac distances. **E.** Unweighted UniFrac distances of the free-foraging lifestyles (Sc, Sw, and Wi) to the Cc group. **F.** SIMPER dissimilaritis of the free-foraging lifestyles (Sc, Sw, and Wi) to the Cc group. PCo, principal coordinate; SIMPER, similarity percentage.

By principal coordinate analysis (PCoA) based on Bray–Curtis distances at the OTU level, intestinal bacterial communities clustered distinctly by different lifestyles (PERMANOVA; *R*^2^ = 0.3017, *P* = 0.001), with clear separation of bacterial communities in multidimensional space (Figure 2A). These results indicate that the gut microbial composition differs significantly among the four lifestyles, irrespective of the evolutionary relationships among the microbial taxa.

Considering the evolutionary relationships and microbial abundance information, although the composition of the microbiota was significantly distinguished among lifestyles by PCoA based on weighted UniFrac distances (PERMANOVA; *R*^2^ = 0.3391, *P* = 0.0010; Figure 2B), weighted UniFrac distances between the free-foraging lifestyles (Sc, Sw, and Wi) and the Cc lifestyle were not significantly different (Kruskal−Wallis test, *P* = 0.59; Figure 2C). This suggests that the lifestyle gradient may not promote evolutionary dissimilarity in the gut microbiota of AEs.

Moreover, we used unweighted UniFrac distances for a PCoA of the AE gut microflora across the four lifestyles, focusing on the presence or absence of microbial taxa (*i.e.*, ignoring abundance information). There were significant differences in microbial composition according to lifestyle (*R*^2^ = 0.2774, *P* = 0.0010). However, lifestyles involving captivity (Cc, Sc, and Sw) clustered with each other, *i.e.*, separately from the Wi lifestyle (Figure 2D). Lifestyles explained 19.71% of the variance along PCoA dimension 1 (PCo1) in comparison to 9.41% of the variance along PCo2. Unweighted UniFrac distances between free-foraging lifestyles (Sc, Sw, and Wi) and the Cc lifestyle significantly increased with the degree of the wild lifestyle (Kruskal−Wallis test, *P* = 1.3E−4; Figure 2E). The similarity percentage (SIMPER) [19] dissimilarity between free-foraging lifestyles (in the order of Sc, Sw, and Wi) and the Cc lifestyle at the OTU level clearly increased (Figure 2F). These results suggest that lifestyles involving artificial feeding might lead to the loss of many bacterial taxa.

### Food types significantly affected the enrichment of intestinal bacteria in AEs

**Figure 3:**
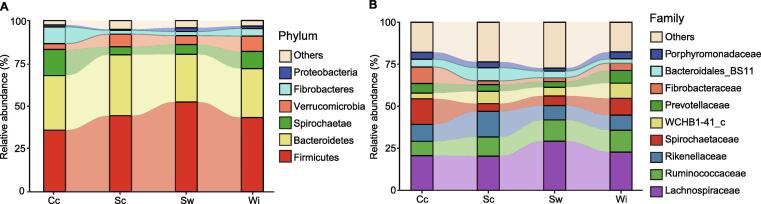
**Taxonomic analysis of the gut microbiomesof AEsacross the lifestyle gradient** **A.** Taxonomic analysis of the gut microbiomes of AEs at the phylum level. **B.** Taxonomic analysis of the gut microbiomes of AEs at the family level.

The results of the taxonomic analysis at the phylum level are shown in Figure 3A. Based on SIMPER analysis, the difference-contributing species at the phylum level among the four lifestyles were Firmicutes, Fibrobacteres, and Proteobacteria. Firmicutes was significantly enriched in the Sw group (Wilcoxon-Mann-Whitney test, *P* < 0.05; Figure S3A), Fibrobacteres was significantly enriched in the Cc group (Wilcoxon-Mann-Whitney test, *P* < 0.05; Figure S3B), and the abundance of Proteobacteria in the Sc group was significantly lower than that in the others (Wilcoxon-Mann-Whitney test, *P* < 0.05; Figure S3C). The difference-contributing species at the family level among the four lifestyles were Lachnospiraceae, Spirochaetaceae, Rikenellaceae, Fibrobacteraceae, Ruminococcaceae, Prevotellaceae, Bacteroidales_BS11, Porphyromonadaceae, and WCHB1-41_c (Figure 3B). The abundance of Ruminococcaceae displayed a decreasing trend from the Wi to Cc group (Figure 3B). Lachnospiraceae was significantly enriched in the Sw group (Wilcoxon-Mann-Whitney test, *P* < 0.05; Figure S3D). Spirochaetaceae and Fibrobacteraceae had the highest abundance in the Cc group (Wilcoxon-Mann-Whitney test, *P* < 0.05; Figure S3E and F). These results suggest that Lachnospiraceae and Ruminococcaceae may be bacterial taxa enriched in the Sw, Sc, and Wi groups due to feeding on natural foods, whereas the high abundance of Spirochaetaceae and Fibrobacteraceae in the Cc group may be related to the high-fiber content in the diet of Cc elephants.

### Abundance variation of bacterial biomarkers in captive and wild groups along the lifestyle gradient

**Figure 4:**
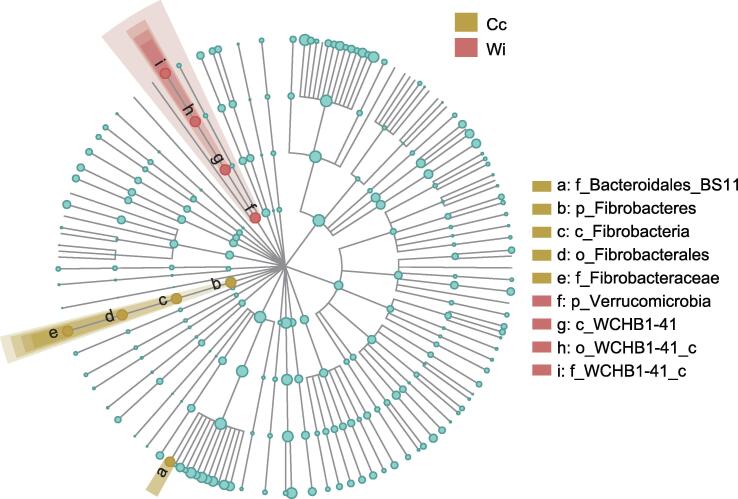
**Bacterial biomarker analysis using linear discriminant analysis effect size**

Based on linear discriminant analysis (LDA) effect size (LEfSe) analysis, the Fibrobacteraceae and Bacteroidales_BS11 families were biomarker bacterial taxa in the Cc group, and the WCHB1-41_c family in the Verrucomicrobia phylum was the biomarker bacterial taxon in the Wi group (Figure 4). Bacterial composition analysis at the phylum level showed that the relative abundance of Verrucomicrobia increased from the Cc, Sw, and Sc groups to the Wi group, and Fibrobacteres in the Cc group was significantly far richer than that in the Sc, Sw, and Wi groups (Wilcoxon-Mann-Whitney test, *P* < 0.05; Figure 3A, Figure S3B).

### Bacterial taxa associated with lifestyle changes

**Figure 5:**
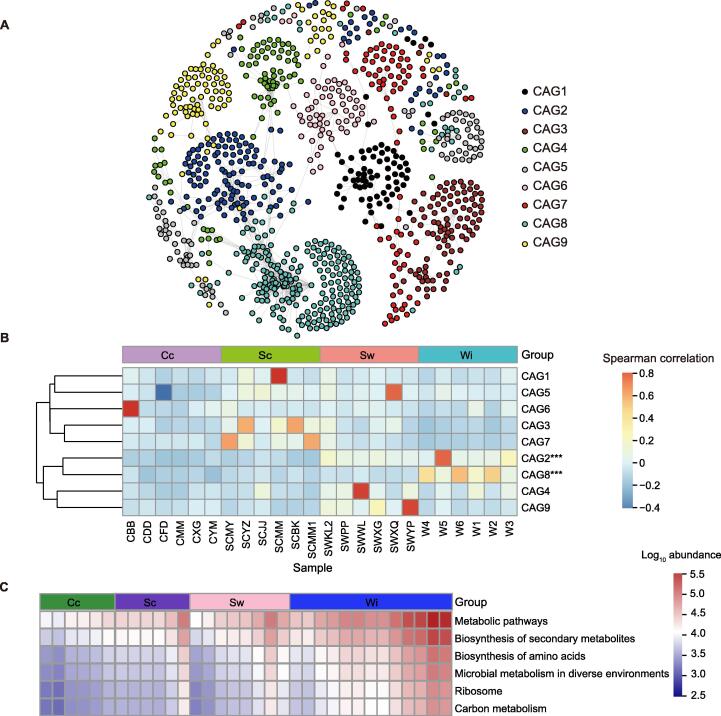
**Cooccurrence network analysis and function prediction** **A.** Nine covariation in CAGs obtained using WGCNA. Dots represent OTUs. **B.** Spearman correlation analysis of the nine CAGs and the four lifestyles. ***, *P* < 0.001 (Kruskal−Wallis test). **C.** Heatmap of functional prediction of WCHB1-41_c by PICRUSt2. WGCNA, weighted correlation network analysis; CAG, coabundance group; OTU, operational taxonomic unit.

At the phylum level, there were clear differences in the abundance of gut bacteria among the four lifestyles (Figure 3A). Therefore, to identify bacterial communities related to lifestyle changes, we performed weighted correlation network analysis (WGCNA) and obtained nine coabundance groups (CAGs) (Figure 5A). Spearman correlation analysis for the relationships between the nine CAGs (CAG1–CAG9) and the four lifestyles showed that CAG2 and CAG8 were significantly related to lifestyle changes (Kruskal−Wallis test, *P* < 0.001; Figure 5B). Further analysis showed that the top ten OTUs most highly correlated with lifestyle changes in CAG2 displayed increased abundances from Cc to Wi lifestyles (Figure S4A). Eight of the top ten OTUs were mainly assigned to the Lachnospiraceae family (OTU1505, OTU496, OTU554, OTU1540, OTU1338, OTU1766, and OTU1799) and the Christensenellaceae family (OTU604) of the Firmicutes phylum, whereas the other two were assigned to the Rikenellaceae family (OTU103) of the Bacteroidetes phylum and the Coriobacteriaceae family (OTU1516) of the Actinobacteria phylum (Figure S4A). However, the top ten OTUs most highly related to lifestyle changes in CAG8 were significantly enriched in the Wi lifestyle (Kruskal−Wallis test, *P* < 0.001; Figure S4B). The significantly enriched bacterial OTUs in CAG8 were mainly from the WCHB1-41_c family (OTU1872, OTU1957, OTU1855, OTU1994, OTU1755, OTU1465, and OTU1470) of the Verrucomicrobia phylum, and the other three OTUs were assigned to the Rikenellaceae family (*RC9*; OTU2011) and the Prevotellaceae family (OTU2036) of the Bacteroidetes phylum and the Ruminococcaceae (*UCG-010*; OTU51) family in the Firmicutes phylum (Figure S4B).

Based on the results of function prediction by PICRUSt2 [20], the main functions of WCHB1-41_c significantly related to lifestyle variation were metabolic pathways, biosynthesis of secondary metabolites, biosynthesis of amino acids, microbial metabolism in diverse environments, ribosome, and carbon metabolism. A heatmap of the functional prediction results showed that the aforementioned functions gradually increased from completely captive to wild populations (Figure 5C).

### Health consequences of lifestyle-related differences in the gut microbiome

**Figure 6:**
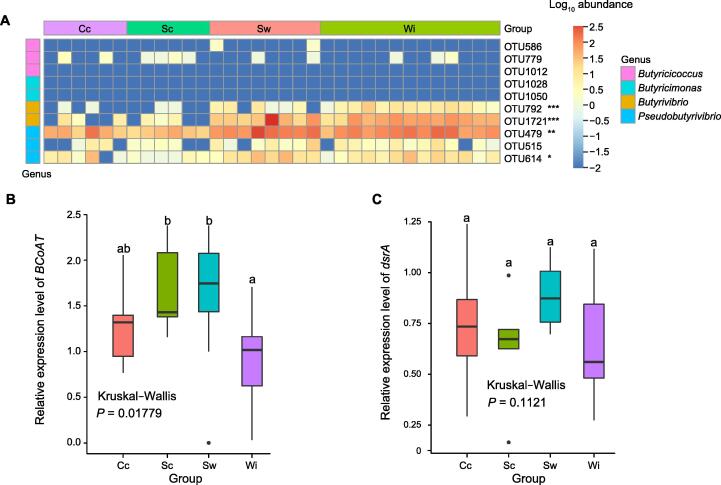
**Content of functional bacteria and genes** **A.** Heatmap analysis of butyrate-producing bacterial OTUs in the gut microbiomes of AEs across four lifestyles determined using 16S rRNA gene sequencing. **B.** Relative expression level of *BCoAT* detected by qRT-PCR. **C.** Relative expression level of *dsrA* detected by qRT-PCR. 16S rRNA gene was used as the internal control. Different letters on the boxes indicate significant differences among groups (Wilcoxon-Mann-Whitney test, *P* < 0.05). *BCoAT*, butyryl-CoA:acetate CoA transferase; *dsrA*, dissimilatory sulfite reductase subunit A.

Reduced gut microbiome diversity in individuals with restricted lifestyles seems to affect the stability of the gut microbiome and may affect host health, and intestinal diseases in captive AEs have previously been reported  [21], [22], [23]. Obvious pathogenic bacteria are rare, but it is possible that changes in the gut microbiome lead to reduced resistance to pathogen interference or invasion. Changes in microbial taxa and metabolic function genes in different environments demonstrate that gut microbiome composition shifts influence howler health [14]. Butyrate is the main source of energy for colon epithelial cells and is thought to have a number of health-promoting benefits [24], [25]. To assess the implications of lifestyle differences for animal health, we analyzed the abundance of butyrate-producing gut bacteria. Although butyrate-producing bacterial taxa were not abundant in the intestines of AEs, the abundances of *Butyrivibrio* (OTU792 and OTU1721) and *Pseudobutyrivibrio* (OTU479 and OTU614) displayed a tendency of clear increase from the Cc to Sc to Sw groups and then slight decrease in the Wi group compared to the Sw group (Kruskal−Wallis test, *P* < 0.05; Figure 6A). Moreover, the abundances of butyrate-producing *Butyricicoccus* (OTU586, OTU779, and OTU1012) were extremely low in the intestines of AEs (Figure 6A). Therefore, butyrate-producing bacteria in the gut of AEs consisted mainly of *Butyrivibrio* and *Pseudobutyrivibrio*. In addition, butyrate-oxidizing bacteria can consume butyrate produced by butyrate-producing bacteria. However, we did not detect the butyrate-oxidizing bacteria *Butyricimonas* (OTU1028 and OTU1050) in the gut microbes of AEs (Figure 6A).

Butyryl-CoA:acetate CoA transferase (BCoAT) is responsible for the final step of butyrate synthesis [26]. qRT-PCR analysis showed that the average relative expression levels of *BCoAT* in individuals with Cc, Sc, Sw, and Wi lifestyles were 1.29, 1.67, 1.80, and 1.05, respectively (Table S4), revealing a gradual decrease from the Sw and Sc groups to the Cc group (Figure 6B). There were significant differences in relative *BCoAT* levels among the four lifestyle groups (Kruskal–Wallis test, *P* = 0.01779; Figure 6B). However, the relative *BCoAT* level in the Wi group was significantly lower than those in the Sc and Sw groups (Wilcoxon-Mann-Whitney test, *P* < 0.01; Figure 6B). These results combined with the dominant species composition (including food-digesting bacteria) analysis (Figure 3, Figure S3) could give important indications of the health consequences of AEs with different lifestyles.

Dissimilatory sulfite reductase subunit A (dsrA) is a key enzyme in sulfate reduction [27]. The average relative expression levels of *dsrA* detected by qRT-PCR in Cc, Sc, Sw, and Wi elephants were 0.74, 0.67, 0.88, and 0.64, respectively (Table S5), There was no apparent trend in the levels of *dsrA* across the lifestyle gradient. The relative levels of *dsrA* did not differ significantly among the four lifestyle groups (Kruskal–Wallis test, *P* > 0.05; Figure 6C), indicating no significant difference in the ability of sulfate-reducing bacteria to produce hydrogen sulfide among the four groups.

## Discussion

By focusing on AEs with distinct lifestyles and diets (including differences in CP and NDF contents) living in sympatry, we illuminated how long-term sustained lifestyle differences influence the gut microbiome in the absence of confounding geographic variation. We found that the gut bacterial community diversity of AEs decreased significantly with the degree of captivity. The gut microbiome is dynamic and responds to subtle dietary changes [28], [29]. Therefore, microbial variation among individuals from the same population is usually interpreted in the context of dietary variation  [5], [30]. In this work, plant species foraged by AEs decreased substantially along the AE lifestyle gradient, from wild to captive lifestyles. Diets containing high-fiber and more plant-based foods should generate a greater diversity of microbial niches than other diet forms [31]. However, the fiber content (*i.e.*, the NDF content) of the main foods increased from the wild to the captive lifestyle (Table S3). Accordingly, the main determinant of the decrease in gut bacterial diversity with the degree of captivity is the decrease in plant species foraged [32], [33], rather than the variation in the fiber content of the diet.

The shared features of individuals with the three lifestyles (Sc, Sw, and Wi) include partial or complete foraging on fresh natural plants and exposure to wild conditions. Schmidt et al. reported that the gut microbiome of deer mice was more similar among neighbors in the same natural environment, regardless of where an individual was born, because fresh natural foods and the wild environment can replenish the gut microbiota [34]. Therefore, fresh natural foods obtained in the wild are able to promote the convergent enrichment of AE gut bacteria. Furthermore, diets (including groundwater), artificial housing environments, and other anthropogenic contacts are shared among individuals in captivity. Artificial feeding with stale plants will cause the loss of gut microbes due to delays in the establishment of an anaerobic environment and the enrichment of anaerobic microbes [35]. Moreover, the hormone and immune stress response caused by human contact or environmental conditions can seriously affect the gut microbiota  [36], [37]. A study has also shown that anthropogenic interference leads to gut microbiome dysbiosis in AEs [38]. These findings may explain why the PCoA showed separation between Wi individuals and other groups. Therefore, the degrees of free foraging and captivity may be the key factors determining the differences in the gut microbial composition among the AEs with four different lifestyles.

Fibrobacteraceae was significantly enriched in Cc populations, which may have been related to the high-fiber content in their diets, because Fibrobacteraceae is the main bacterial taxon that degrades cellulose [39]. Bacteroidales_BS11 was significantly enriched in the Sc populations, enabling AEs to nutritionally adapt to rapidly changing feed in captivity and free-foraging states  [40]. Lachnospiraceae was significantly enriched in the Sw group, which can affect the host by promoting resistance to colonization by intestinal pathogens [41]. Consequently, the increase in the abundance of Lachnospiraceae may be beneficial for immunological processes in individuals with the free-foraging lifestyle.

The combined results of the SIMPER, LEfSe, and WGCNA analyses showed that the bacterial taxon WCHB1-41_c was significantly affected by lifestyle variations. From the perspective of abundance change, WCHB1-41_c was gradually enriched in AEs due to free foraging. This bacterial taxon may also gradually be lost from AEs with an increasing degree of captivity. Guo et al. found that uncultured *Eubacterium* WCHB1-41_c played a vital role in regulating nutritional requirements under extreme environmental conditions with sparse forage. A shift in the gut microbiome of yaks was observed in response to dietary changes in the harsh cold season to effectively utilize nitrogen and energy [42]. Wei et al. reported that early weaned yak calves had high mortality due to a lack of nutrition and harsh environmental conditions [43]. They conducted a supplementary feeding trial of *Astragalus* root extract on weaned yak calves and found that the proportion of WCHB1-41_c showed an increasing trend with an increase in *Astragalus* root extract supplementation. A growth performance test of yak calves showed that their final bodyweight and average daily gain (ADG) were significantly higher than those of the control, and the dry matter intake (DMI)/ADG ratio was significantly lower in calves with *Astragalus* root extract supplementation than in the control calves. The concentrations of serum immunoglobulin G (IgG) and interleukin-2 (IL-2) in calves fed *Astragalus* root extract were higher than those in the control group. This indicated that *Astragalus* root extract caused an increase in the proportion of WCHB1-41_c bacterial taxa to improve the body’s immunity against harsh environments [43]. Therefore, the enrichment of uncultured *Eubacterium* WCHB1-41_c in WAEs in this study indicates that the habitat of WAEs may not be optimal, and the scarcity of forage may induce this issue. In turn, the enrichment of uncultured *Eubacterium* WCHB1-41_c may be beneficial for WAEs to cope with harsh conditions in the wild.

Based on our observational field study of endangered WAEs, we cannot establish a causal relationship between the lifestyle and health status of this animal. However, our results provide some evidence that the Wi lifestyle may negatively affect host health via diet-associated shifts in the gut microbiome. In general, butyrate promotes intestinal development and health [44], [45], while sulfate-reducing bacteria utilize hydrogen less efficiently, and their final metabolic product H_2_S can cause carcinogenesis and inflammation [46], [47]. In our study, although the relative expression level of *dsrA* did not differ significantly among the four lifestyles, the relative expression level of *BCoAT* showed an increasing trend from Cc to Sw individuals. However, the relative *BCoAT* level in Wi individuals was almost as low as that in Cc individuals (Figure 6B), indicating that the Wi lifestyle involved unfavorable factors for health. Further studies on these unfavorable factors (reduced resource availability, such as food and habitat) associated with the Wi lifestyle can also assist in exploring the factors contributing to the northward migration of WAEs.

Although the results of this study indicate that plant diversity in the diets associated with the four lifestyles drives the patterns of microbiome diversity, further studies are needed to determine whether dietary diversity or specific plant metabolites that differ among plant species promote a stable gut microbiome composition with high alpha diversity [14]. Plant *trnL* (UAA) and bacterial 16S rRNA gene sequencing analyses can be used to more accurately determine the relationship between the dietary composition and gut microbiome of AEs with different lifestyles [42]. In addition, studies examining the relationship between the gut microbiome composition and the health of AEs by directly measuring the factors affected by the microbiome, such as glucocorticoid levels and immunoglobulin A levels, should be carried out [48], [49], and the abundance and diversity of intestinal pathogens [50], parasites [51], and viruses [52] should be determined by metagenomic sequencing.

## Conclusion

Our results indicate that the reduced microbiome diversity of captive AEs is an established fact, whereas increased unfavorable factors (reduced resource availability, such as food and habitat) is an issue affecting WAEs. This study provides a basis for developing new conservation techniques and tools to better assess the effects of human activity on WAEs health and the challenges faced by animal populations that are forced to migrate.

## Materials and methods

### Sample description and fecal sample collection

**Figure 7:**
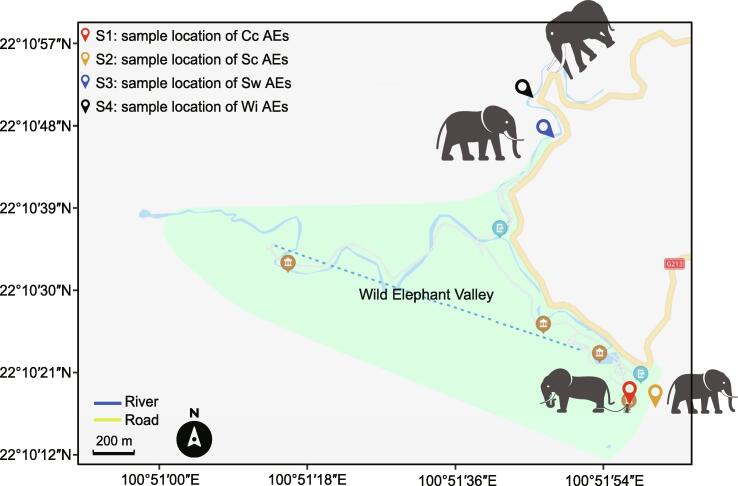
**Map of the sampling sites** Geographical distribution of all sampling sites in the Wild Elephant Valley and nearby forest.

All AEs studied herein live in the Wild Elephant Valley in Xishuangbanna, China (Figure 7), located in a tropical climate zone with dry and wet seasons. The annual temperature is 18 °C–30 °C, and the annual relative humidity is approximately 80%. Four AE lifestyles have existed in the Wild Elephant Valley for a long time. First, animals in the Cc group are present in the scenic area (sampling S1 in Figure 7). These include long-term captive elephants and their offspring. Their diet is elephant grass (*Pennisetum purpureum*) supplemented with sugarcane and carrots. The second is the Sc group, which involves interactions with tourists; these animals can graze or forage for natural foods in the wild and are fed elephant grass by the staff in the scenic area (sampling S2 in Figure 7) at other times. The third is the Sw group, which includes abandoned calves, rescued elephants, and their offspring. As their survivability is poor, they are accompanied by a breeder to forage in the wild and to prevent individuals from being lost or attacked by wild elephants. At night, they return to the Breeding and Rescue Center (sampling S3 in Figure 7), approximately 1.4 km from the areas where the aforementioned two types of AEs live. The fourth was the Wi group.

To maintain consistency in sampling conditions, fecal samples of all AEs were collected under similar environmental conditions during the dry season (March 2019; Table S6). Fresh feces were collected from 20 healthy AEs that did not receive antibiotics (out of a total of 42 AEs in the scenic area and breeding center) and 13 wild individuals. In total, 33 AE individuals were included (*n* = 6, 6, 8, and 13 for Cc, Sc, Sw, and Wi lifestyles, respectively). Detailed information about AEs was obtained from the breeders and our observations (Table S6). The detailed sampling method was as follows [53]: Cc, Sc, and Sw individuals were accompanied by the breeders until defecation, and samples were collected immediately from the center of fresh feces with sterile tweezers, placed in sterile centrifuge tubes, and stored in liquid nitrogen. In the case of WAEs, we drove to site S4 (Figure 7) after receiving information from AE monitors and observed the animals until they left. Only fresh fecal samples that were deposited within half an hour of an animal’s departure were collected, transported in liquid nitrogen, and then stored at –80 °C until DNA extraction.

Although the sex ratio of each group was highly unbalanced owing to the scarcity of samples, three female and three male adult AEs in the Sw group were selected to examine the influence of sex on gut microbial diversity (Table S6). Similarly, samples from three juveniles (4 years old; one from the Sc group and two from the Sw group) and six adults (20–33 years old; all from the Sw group) were selected to analyze the influence of age on the gut microbiome (Table S6).

### Identification of the foraging habits of AEs

Food for Cc elephants was conspicuously stocked next to the elephant house at site S1 (Figure 7). However, Sc and Sw elephants need to be constantly followed to observe and record the types of wild plants consumed. Image data and plant specimens were collected to aid in the identification and classification of the most frequently eaten plant species. Because WAEs are aggressive, the foraging survey was completed with the help of forest rangers and AE monitors. Using the plant species identification and verification methods reported by Jiang et al. [54], a new list of fed/foraged plants was generated for individuals under different lifestyles in this study (Table S1).

### DNA extraction, gene amplification, and sequencing

Microbial genomic DNA was extracted from 33 fecal samples using the EZNA Soil DNA Kit (Catalog No. D5625-01, Omega, Irving, TX). DNA quality and quantity were evaluated using 1% agarose gel electrophoresis and a NanoDrop 2000 spectrophotometer, respectively (ThermoFisher Scientific, Waltham, MA). The hypervariable region V3–V4 of the bacterial 16S rRNA gene was amplified with the primer pair 338F (5′-ACTCCTACGGGAGGCAGCAG-3′) and 806R (5′-GGACTACHVGGGTWTCTAAT-3′) using an ABI GeneAmp 9700 PCR thermocycler (ThermoFisher Scientific). The PCR mixtures contained 4 μl of 5× TransStart FastPfu Buffer (Catalog No. AP221-01/11, TransGen Biotech, Beijing, China), 2 μl of 2.5 mM dNTPs, 0.8 μl each of 5 μM forward and reverse primers, 0.4 μl of TransStart FastPfu DNA Polymerase (Catalog No. AP221-01/11, TransGen Biotech), 10 ng of template DNA, and ddH_2_O up to 20 μl. PCR amplification was performed in triplicate under the following conditions: 95 °C for 3 min, followed by 30 cycles of 95 °C for 30 s, 55 °C for 30 s, and 72 °C for 45 s, and a final extension at 72 °C for 10 min. Purified amplicons were pooled in equimolar aliquots and then sequenced on the Illumina MiSeq platform (Illumina, San Diego, CA) to obtain paired-end reads [55].

### Processing of sequencing data

Raw 16S rRNA gene sequencing reads were demultiplexed and quality-filtered using fastp (version 0.20.0) [56] and then merged using FLASH (version 1.2.7) [57]. Stringent criteria were established for quality. Pair reads in length of 300 bp were truncated at any site that received an average quality score < 20 over a 50-bp sliding window. Truncated reads shorter than 50 bp and reads with ambiguous characters were discarded. Sequences required an overlap larger than 10 bp for assembly, and the maximum mismatch ratio of the overlap region was 0.2. Reads that could not be assembled were discarded. Samples were distinguished by barcodes and primers, and the sequence direction was adjusted accordingly. Exact barcode matching was required, and a mismatch of two nucleotides in primer matching was allowed.

OTUs with a 97% similarity cutoff [58], [59] were clustered using UPARSE (version 7.1) [58]; chimeric sequences were identified and removed. Taxon assignments for each representative OTU sequence were determined using RDP classifier (version 2.2) [60] with the 16S rRNA gene database (Silva version 138) with a confidence threshold of 0.7.

### Cooccurrence network analysis

To identify correlated OTUs from the intestinal bacteria of AEs with the four lifestyles, we performed a cooccurrence network analysis at the OTU level. A minimum number of sequences was randomly extracted from each sample to obtain the OTU table; 967 OTUs were selected for which the abundance was not 0 in at least 50% of the samples for the WGCNA. The WGCNA R package (version 1.72-1) [61], [62] was then used to analyze OTUs with sequence reads in at least 12 samples. The soft-thresholding power was set to 5, and the scale-free topology model fit index *R*^2^ was approximately 0.9. The “softConnectivity” function was used to check the scale-free topology (scale *R*^2^ = 0.84, slope = –2.29), and the “adjacency” function was used to obtain the adjacency matrix (power = 4, type = “unsigned”, other parameters default). The “TOMsimilarity” function was used to convert the adjacency matrix into a topological overlap matrix (TOM). After using the TOM to cluster OTUs, the modules (also denoted as subnetworks) were produced using a dynamic tree to cluster OTUs into modules (minimum module size was 60 OTUs). Modules with a Spearman correlation coefficient exceeding 0.7 were merged. The “corAndPvalue” function was used to calculate the correlation between each module and lifestyle to obtain considerable modules related to lifestyle variation.

### Alpha diversity, beta diversity, and Venn analyses

The OTU table was rarefied to equal sequence number (*n* = 24,600) for each sample with 1000 replicates (Table S7). A rarefaction curve was generated using the vegan package (version 2.5-7) in R (https://github.com/vegandevs/vegan/releases/tag/v2.5-7). Alpha diversity indices were calculated using MOTHUR (version 1.30.1) [63]. Kruskal–Wallis test was used to detect the significant difference between multiple groups, and Wilcoxon-Mann-Whitney test was used to analyze the significant difference between two groups. *P* < 0.05 was considered statistically significant.

The vegan package was used to calculate beta diversity and the Bray–Curtis similarity matrix. Considering the evolutionary relationships among microorganisms, beta diversity was estimated by computing the unweighted and weighted UniFrac distances and visualized by PCoA, and the results were plotted using the GUniFrac (version 1.6) and ape (version 5.5) packages in R [64], [65]. PERMANOVA (adonis, permutations = 999) was performed to evaluate differences in beta diversity between two groups. To determine the contribution of various bacterial taxa to dissimilarity in microbial communities along a lifestyle gradient, the SIMPER analysis was performed using the vegan R package.

Venn analysis was performed by calling the ggVennDiagram package [66] of R using OTU table data.

### Taxonomic analysis

The LEfSe analysis was performed to find statistically significant biomarkers between Cc and Wi groups. The LDA threshold was set to 4, and the taxonomic level was from the phylum to the family level. A heatmap was generated to visualize the data using the pHeatmap package (version 1.0.12) in R (https://cran.rproject.org/web/packages/pheatmap/).

### qRT-PCR analysis

To verify the butyrate-producing ability of gut bacteria and to determine the impact of lifestyle on the health of AEs, *BCoAT* was selected for qRT-PCR analysis. Moreover, to determine the ability of intestinal sulfate-reducing bacteria to produce harmful hydrogen sulfide, *dsrA* was selected for qRT-PCR analysis according to a method described by Nava and colleagues [67]. The primers BCoATscrF (5′-GCIGAICATTTCACITGGAAYWSITGGCAYATG-3′) and BCoATscrR (5′-CCTGCCTTTGCAATRTCIACRAANGC-3′) were used to quantify the relative expression levels of *BCoAT* of butyrate-producing bacteria in the samples [68]. The primers DSR-F (5′-ACSCACTGGAAGCACGCCGG-3′) and DSR-R (5′-GTGGMRCCGTGCAKRTTGG-3′) were used to determine the relative expression levels of *dsrA* of sulfate-reducing bacteria in the samples  [69]. The reaction mixture contained 5 μl of 1× SYBR Green Supermix (Catalog No. MT0017, Danfeng Biotech, Chengdu, China), 0.5 μl of each primer (200 nM), and 1 μl of template DNA, and ddH_2_O was added to bring the volume to 10 μl. The thermal cycling conditions were as follows: 95 °C for 3 min for initial denaturation; 39 cycles of 95 °C for 10 s and 65 °C for 30 s, and a melting curve analysis (60 °C–95 °C, +1 °C/cycle, holding 4 s). Data were analyzed using the Pfaffl method, based on the 2^−^^ΔΔCt^ method [70], [71] and normalized against the 16S rRNA gene as the housekeeping gene.

## Data availability

Raw sequence data obtained in this study have been deposited in the Genome Sequence Archive [72] at the National Genomics Data Center, Beijing Institute of Genomics, Chinese Academy of Sciences / China National Center for Bioinformation (GSA: CRA007093), and are publicly accessible at https://ngdc.cncb.ac.cn/gsa.

## Competing interests

The authors have declared no competing interests.

### CRediT authorship contribution statement

**Chengbo Zhang:** Conceptualization, Methodology, Formal analysis, Writing – original draft, Software, Data curation, Investigation. **Zhenghan Lian:** Methodology, Software, Visualization. **Bo Xu:** Conceptualization, Resources. **Qingzhong Shen:** Resources, Investigation. **Mingwei Bao:** Resources, Investigation. **Zunxi Huang:** Conceptualization, Writing – original draft, Writing – review & editing, Supervision. **Hongchen Jiang:** Writing – review & editing. **Wenjun Li:** Writing – review & editing, Supervision. All authors have read and approved the final manuscript.

## Supplementary material

Supplementary Figure S1 **Rarefaction curves** Sample-based rarefaction curves showing the increase in OTU abundance as a function of the number of reads sampled. Each curve represents the rarefaction curve of one individual sample (n = 33). OTU, operational taxonomic units.

Supplementary Figure S2 **Effects of gender and age on intestinal microbiota of Asian elephant A.** Chao 1 and Shannon indices between male and female Asian elephants at the OTU level. **B.** PCoA of the gut microbial composition between sexes. **C.** Chao 1 and Shannon indices between juveniles and adults at the OTU level. **D.** PCoA of the gut microbial composition between juveniles and adults. PCoA, principal coordinate analysis.

Supplementary Figure S3 **Abundance of the difference-****contributing species at the phylum****and family****levels****among the four lifestyles****A.** Abundance difference analysis in the phylum Firmicutes. **B.** Abundance difference analysis in the phylum Fibrobacteres. **C.** Abundance difference analysis in the phylum Proteobacteria. **D.** Abundance difference analysis in the family Lachnospiraceae. **E****.** Abundance difference analysis in the family Spirochaetaceae. **F.** Abundance difference analysis in the family Fibrobacteraceae. *, *P* < 0.05; **, *P* < 0.01; n.s., not significant (Wilcoxon-Mann-Whitney test).

Supplementary Figure S4 **Abundance of the top 10 OTUs in CAG2 and CAG8** Abundance heatmap analysis of the top 10 OTUs related to lifestyle variations in CAG2 (**A**) and CAG8 (**B**).

Supplementary Table S1 **Checklist of plants fed/foraged by the Asian elephants in China**

Supplementary Table S2 **CP and NDF content of the main foraging plants consumed by Asian elephants**

Supplementary Table S3 **Average CP and average NDF content of the main foraging plants consumed by Asian elephants in four lifestyle gradients**

Supplementary Table S4 **Relative quantitative PCR analysis results of *BCoAT***

Supplementary Table S5 **Relative quantitative PCR analysis results of *dsrA***

Supplementary Table S6 **Details of the Asian elephant included in the study**

Supplementary Table S7 **The number of effective sequences and OTU numbers obtained in each sample**
