# Supplementary figures and images for: Gut Microbiome Variation Along A Lifestyle Gradient Reveals Threats Faced by Asian Elephants
Source: Genomics Proteomics Bioinformatics. 2023 Apr 22;21(1):150–63. doi: 10.1016/j.gpb.2023.04.003 (PMC10372918; doi:10.1016/j.gpb.2023.04.003)

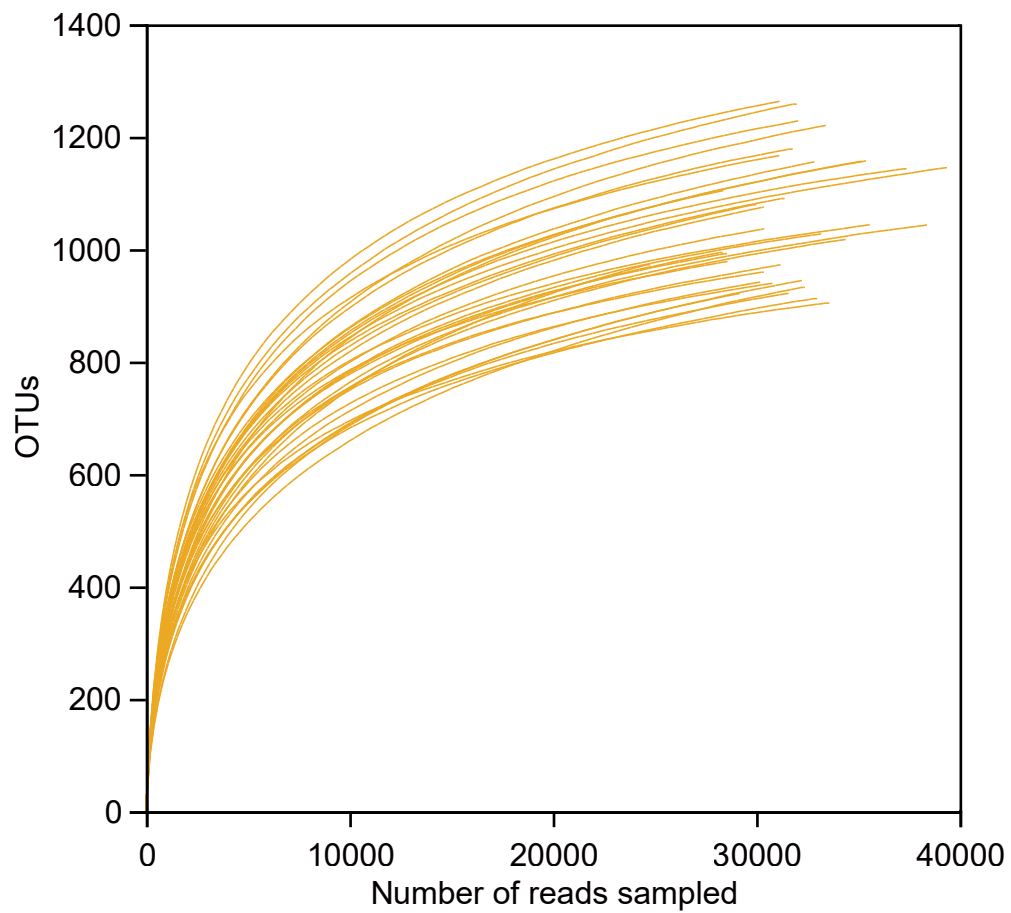

Supplement: Supplementary Figure S1 — Rarefaction curves Sample-based rarefaction curves showing the increase in OTU abundance as a function of the number of reads sampled. Each curve represents the rarefaction curve of one individual sample (n = 33). OTU, operational taxonomic units. [file mmc1.pdf]

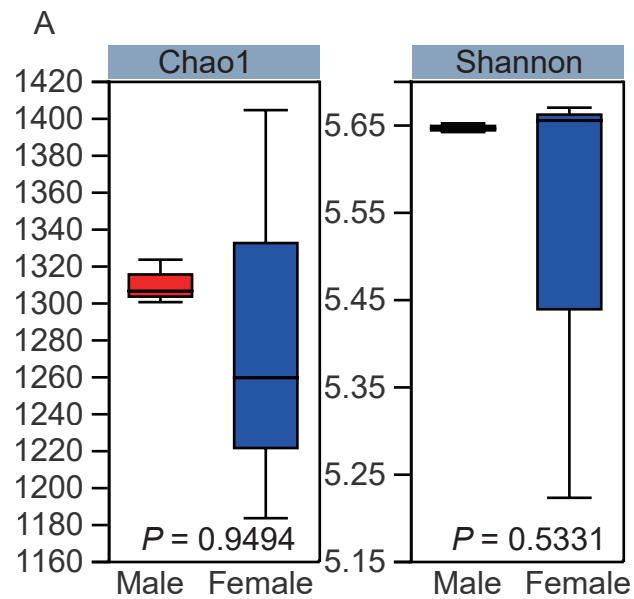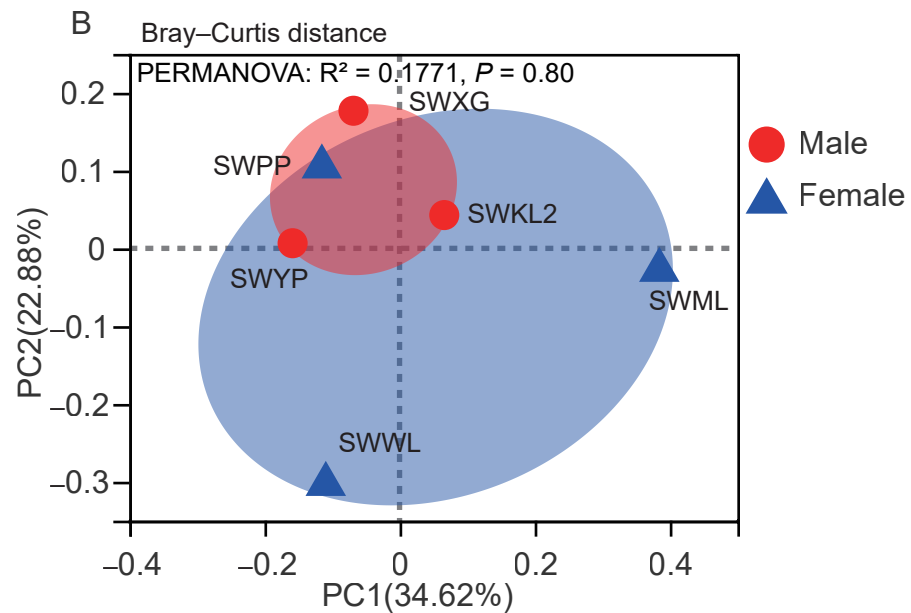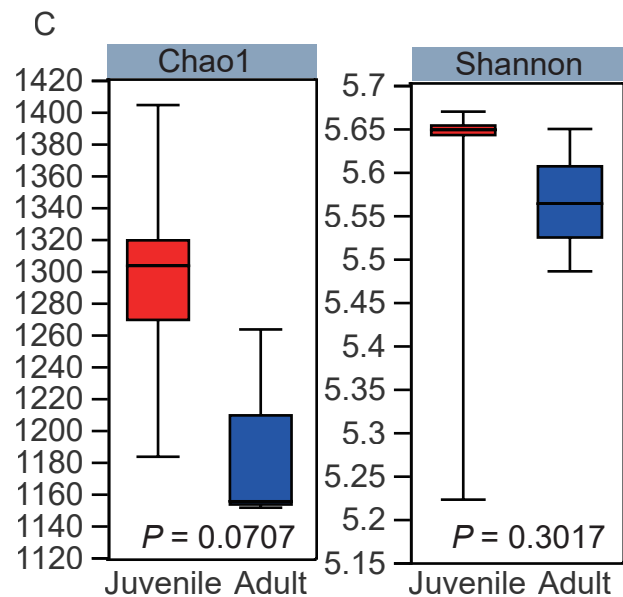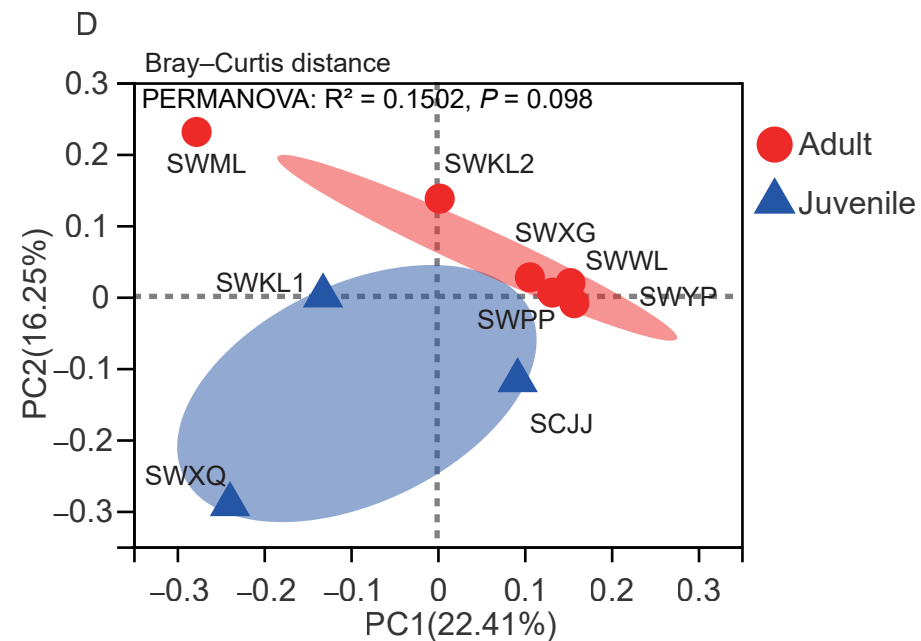

Supplement: Supplementary Figure S2 — Effects of gender and age on intestinal microbiota of Asian elephant A. Chao 1 and Shannon indices between male and female Asian elephants at the OTU level. B. PCoA of the gut microbial composition between sexes. C. Chao 1 and Shannon indices between juveniles and adults at the OTU level. D. PCoA of the gut microbial composition between juveniles and adults. PCoA, principal coordinate analysis. [file mmc2.pdf]

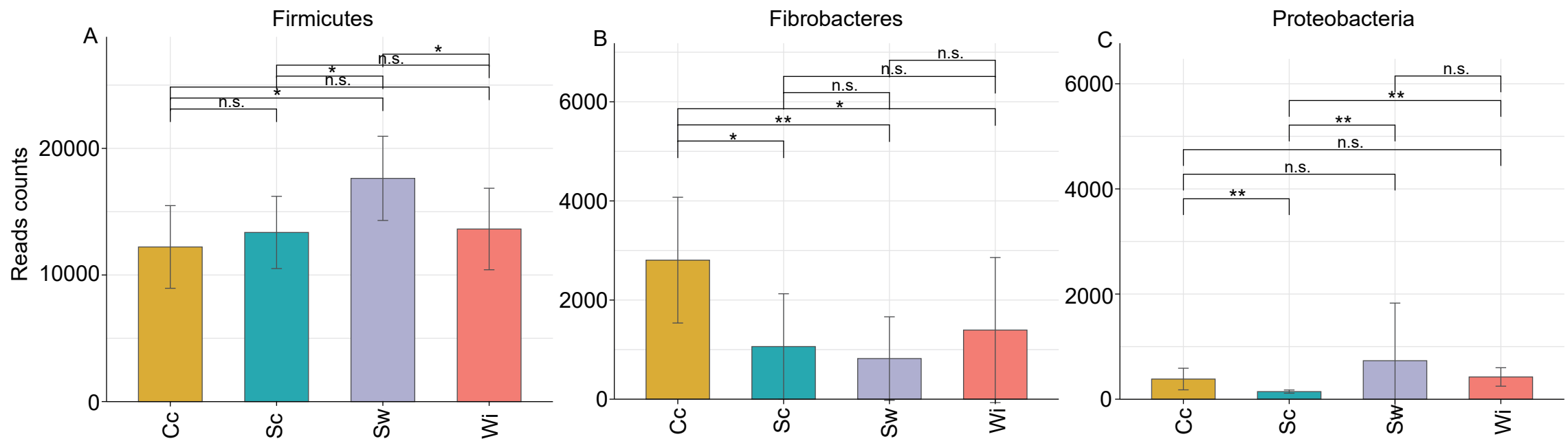

Supplement: Supplementary Figure S3 — Abundance of the difference-contributing species at the phylumand familylevelsamong the four lifestylesA. Abundance difference analysis in the phylum Firmicutes. B. Abundance difference analysis in the phylum Fibrobacteres. C. Abundance difference analysis in the phylum Proteobacteria. D. Abundance difference analysis in the family Lachnospiraceae. E. Abundance difference analysis in the family Spirochaetaceae. F. Abundance difference analysis in the family Fibrobacteraceae. *, P < 0.05; **, P < 0.01; n.s., not significant (Wilcoxon-Mann-Whitney test). [file mmc3.pdf]

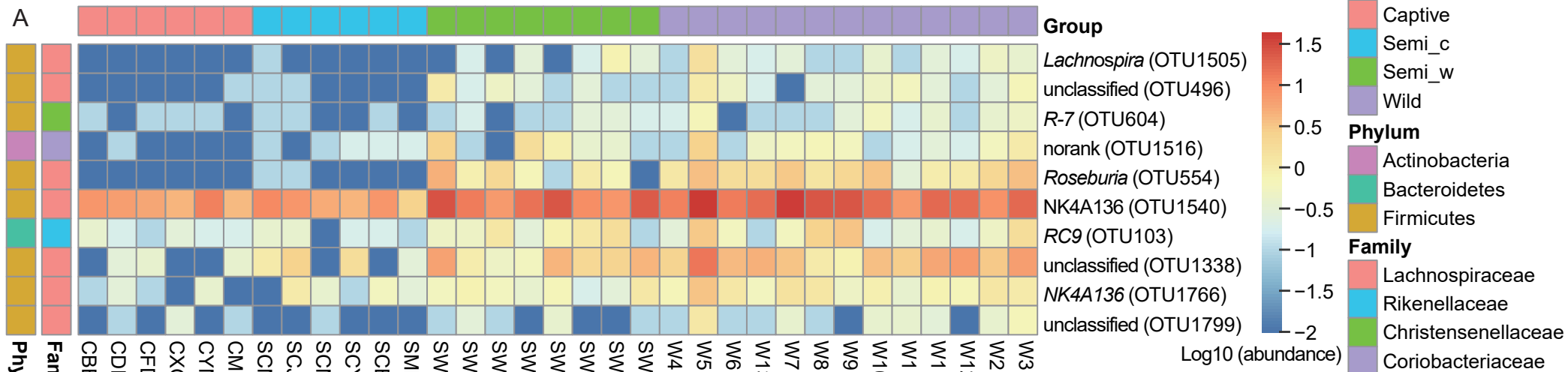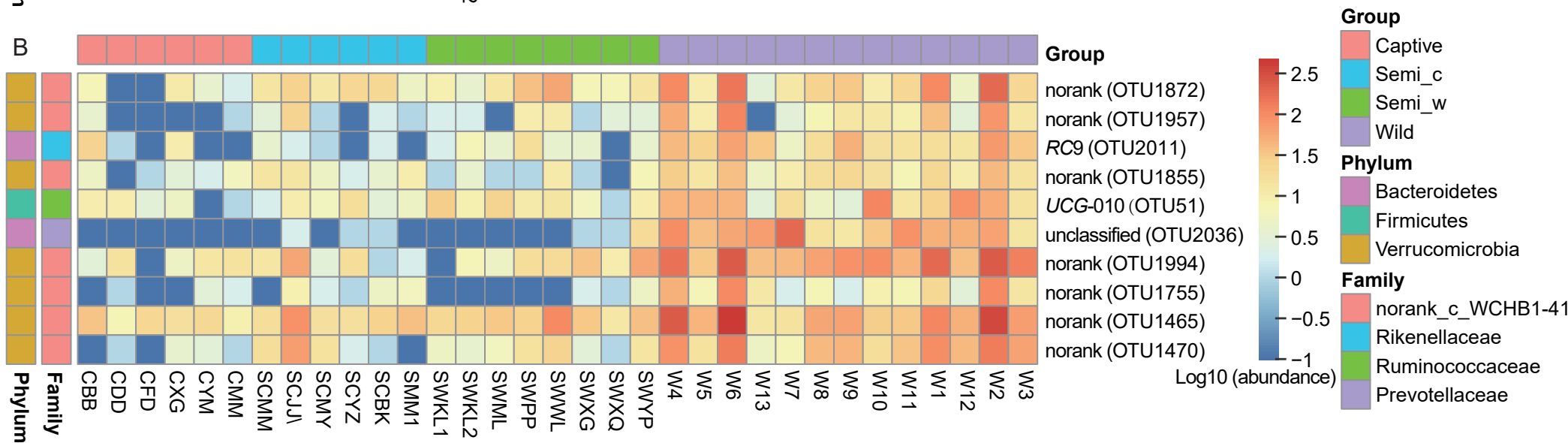

Supplement: Supplementary Figure S4 — Abundance of the top 10 OTUs in CAG2 and CAG8 Abundance heatmap analysis of the top 10 OTUs related to lifestyle variations in CAG2 (A) and CAG8 (B). [file mmc4.pdf]
